# The mediating effect of somatic symptom disorder between psychological factors and quality of life among Chinese breast cancer patients

**DOI:** 10.3389/fpsyt.2023.1076036

**Published:** 2023-05-12

**Authors:** Zimeng Li, Yening Zhang, Ying Pang, Yi He, Lili Song, Yan Wang, Shuangzhi He, Lili Tang

**Affiliations:** Department of Psycho-Oncology, Key Laboratory of Carcinogenesis and Translational Research (Ministry of Education/Beijing), Peking University Cancer Hospital and Institute, Beijing, China

**Keywords:** breast cancer, psycho-oncology, medication effect, quality of life, somatic symptom disorder

## Abstract

**Objective:**

We conducted this cross-sectional study to explore the mediating and predicting role of somatic symptom disorder (SSD) between psychological measures and quality of life (QOL) among Chinese breast cancer patients.

**Methods:**

Breast cancer patients were recruited from three clinics in Beijing. Screening tools included the Patient Health Questionnaire-15 (PHQ-15), the Patient Health Questionnaire-9 (PHQ-9), the General Anxiety Disorder-7 scale (GAD-7), the Health Anxiety Scale (Whiteley Index-8, WI-8), the Somatic Symptom Disorder B-Criteria Scale (SSD-12), the Fear of Cancer Recurrence scale (FCR-4), the Brief Illness Perception Questionnaire (BIPQ-8), and the Functional Assessment of Cancer Therapy-Breast (FACT-B). Chi-square tests, nonparametric tests, mediating effect analysis, and linear regression analysis were used for the data analysis.

**Results:**

Among the 264 participants, 25.0% were screened positive for SSD. The patients with screened positive SSD had a lower performance status, and a greater number of patients with screened positive SSD received traditional Chinese medicine (TCM) (*p* < 0.05). Strong mediating effects of SSD were found between psychological measures and QOL among patients with breast cancer after adjusting for sociodemographic variables as covariates (*p* < 0.001). The range of the percentage mediating effects was 25.67% (independent variable = PHQ-9) to 34.68% (independent variable = WI-8). Screened positive SSD predicted low QOL in physical (B = −0.476, *p* < 0.001), social (B = −0.163, *p* < 0.001), emotional (B = −0.304, *p* < 0.001), and functional (B = −0.283, *p* < 0.001) well-being, as well as substantial concerns caused by breast cancer (B = −0.354, *p* < 0.001).

**Conclusion:**

Screened positive SSD had strong mediating effects between psychological factors and quality of life among breast cancer patients. Additionally, screened positive SSD was a significant predictor of lower QOL among breast cancer patients. Effective psychosocial interventions for improving QOL should consider the prevention and treatment of SSD or integrated SSD caring dimensions for breast cancer patients.

## 1. Introduction

Breast cancer is the most common malignant tumor in women worldwide. According to the latest data, breast cancer ranks first in cancer incidence among Chinese women, and the mortality rate ranks fourth (1, 2). The incidence and mortality of breast cancer in China have been increasing in the past 10 years; therefore, improvements in the quality of life (QOL) of these patients are becoming increasingly important from both clinical and research perspectives. Chinese breast cancer patients are generally younger than patients in developed countries, with the median age at the time of diagnosis of breast cancer in China being approximately 50 years of age, which is 10 years earlier than that in the United States and the European Union (3).

The diagnosis of breast cancer affects the physical and mental health, QOL, and social function of patients, and they often face serious psychological distress and emotional problems, such as anxiety, depression, and fear (4, 5). Moreover, due to the disease itself and treatment-related adverse reactions, various physical and psychological symptoms can result in a considerable symptom burden, which seriously affects patients’ QOL and social functions (6). Studies have shown that the symptom burden of breast cancer patients is closely related to anxiety and other emotional problems (7), and cancer-related somatic symptoms are also affected by physical status, cognition, emotion and other factors (8). Some patients experience somatic symptoms, although these symptoms are not well explained by existing medical diseases. In recent years, the physical symptoms of patients with mental illness have become more and more obvious and diverse, especially the impact of new coronary pneumonia in recent years (9). The Diagnostic and Statistical Manual of Mental Disorders, 5th edition (DSM-5) defines this condition as somatic symptom disorder (SSD), which is characterized by persistent somatic symptoms (lasting for more than 6 months) that are accompanied by excessive thoughts, feelings, and behaviors that are related to the symptoms but disproportionate to the seriousness of the symptoms (10).

Studies have shown that patients with SSD suffer from multiple disturbances of physical and psychological symptoms, which affect their quality of life. The influencing factors of quality of life are multidimensional, including the severity of somatic symptoms, the number of somatic symptoms, and disease cognitions. A high number of somatic symptoms, a high level of somatic symptom disorder and a negative illness perception will significantly reduce the quality of life of cancer patients (11, 12). Li reported the prevalence of and risk factors for SSD among Chinese breast cancer patients by SCID interview, which provided a greater understanding about this issue and aroused our interest in exploring the influence of SSD on quality of life in this group (13). Therefore, we reanalyzed these data to understand the mediating and predicting role of SSD between psychological variables and QOL in Chinese breast cancer patients and to explore the applicability of the SSD-12 as a screening tool in clinical oncology.

## 2. Methods

### 2.1. Participants

A total of 273 participants were recruited from three clinics (two breast cancer clinics and one psychiatric clinic) in two hospitals in Beijing, China, from February 2019 to October 2019. All patients who entered one of the three clinics were informed of this study and invited to participate by research assistants. Eligible patients who came to the clinics were recommended to join the study by the doctors. The patients who agreed to join were asked to fill out the self-reported questionnaires. Participation was voluntary, and the patients signed informed consent forms and agreed to the evaluation and processing of the collected data. This study was approved by the Ethics Committees of Peking University Cancer Hospital (2019YJZ06).

The inclusion criteria included age ≥ 18 years, pathological diagnoses of breast cancer and adequate Chinese reading and writing skills. The exclusion criteria included severe cognitive impairments, psychosis, and acute suicidal tendencies.

### 2.2. Assessment instruments

***The Somatic Symptom Disorder B-Criteria Scale (SSD-12)*** (14) is a self-assessment scale developed from the B criteria for an SSD diagnosis that is used to quantitatively evaluate patients’ feelings and coping styles in relation to physical discomfort. It includes 12 items, and each item is rated from 0 (never) to 4 (frequently), with total scores ranging from 0 to 48. The Chinese version of the SSD-12 has also been verified to have good reliability and validity, and a score of 16 is recommended as the cut-off value (15). The SSD-12 was used to divide the sample into a non-SSD group (score < 16) and an SSD group (score ≥ 16). Although the SSD-12 could not fully meet all diagnosis criteria for SSD, we used the SSD-12 as the main screening tool in this reanalysis study because the SSD-12, as a patient-reported outcome (PRO) measurement, is preferable for psychosocial distress screening in oncology practice and provides valuable referral recommendations to multidisciplinary professionals.

***The Patient Health Questionnaire-15 (PHQ-15)*** (16) was used to evaluate the number of somatic symptoms and the degree of distress experienced in the past 4 weeks. It includes 15 somatic symptoms or symptom clusters. Each item is rated as 0 (no trouble), 1 (few troubles), or 2 (many troubles), with total scores ranging from 0 to 30. The optimal cut-off points of 5, 10, and 15 represented low, medium, and high somatic symptom severity, respectively (16). The Chinese version of the PHQ-15 has good reliability and validity (17).

***The Patient Health Questionnaire-9 (PHQ-9)*** (18) was designed to evaluate the degree of depression experienced in the past 2 weeks. It includes 9 items, and each item is rated from 0 (not at all) to 3 (nearly every day), with total scores ranging from 0 to 27. The Chinese version of the PHQ-9 was validated in outpatients with multiple somatic symptoms, and the optimal cut-off point for moderate depression in Chinese outpatients was 10 (19).

***The General Anxiety Disorder-7 (GAD-7)*** was used to assess anxiety levels experienced in the past 2 weeks. It includes 7 items rated from 0 to 3, with total scores ranging from 0 to 21. The Chinese version of the GAD-7 has been validated in general hospital outpatients, and the cut-off points for mild, moderate, and severe anxiety disorder were 4, 9, and 12, respectively (20).

***The Health Anxiety Scale (Whiteley index-8, WI-8)*** is a brief scale for assessing disease belief and health concern experienced in the past four weeks and includes 8 items. Each item is rated from 1 to 5, with a total score ranging from 8 to 40. Higher scores indicate a higher degree of anxiety (21). The cut-off point for the WI-8 was 19 (22).

***The Fear of Cancer Recurrence (FCR-4)*** includes 4 items, and each item is rated from 0 to 4, with total scores ranging from 0 to 16. Higher scores indicate a greater fear of cancer recurrence, and no cut-off point has been recommended (23). The Chinese version of the FCR-4 has not yet been validated.

***The Brief Illness Perception Questionnaire (BIPQ-8)*** was used to assess the cognitive and emotional representations of patients with regard to their own diseases. It includes the following 8 items: influence, duration, personal control, treatment control, symptom identification, concern, understanding, and emotional response. Each item is rated from 0 to 10. No cut-off value was suggested; higher scores indicate higher degrees of feeling threatened and negative illness perceptions. One study explored the Chinese version of the BIPQ-8 among local cancer patients and proved that the BIPQ-7 (item 7 “how well do you feel you understand your illness” was deleted) had good validity and reliability (24). No further study using this changed version was reported, especially in the breast cancer group in China. We used the original version of the BIPQ-8 in this study.

***The Functional Assessment of Cancer Therapy-Breast (FACT-B)*** was used to evaluate the QOL of breast cancer patients over the past week. It included four subscales and an additional scale with 37 items. The four subscales included physical well-being (PWB), social/family well-being (SWB), emotional well-being (EWB) and functional well-being (FWB). The breast cancer subscale (BCS) was an additional subscale that contained 10 items related to breast cancer. Each item was rated from 0 to 4. Higher scores indicated higher QOL, and no cut-off point was recommended by the development group. The Chinese version of the FACT-B has also been validated in breast cancer patients (25).

All investigators were trained to use the assessment instruments and became competent in conducting consistent evaluations.

### 2.3. Statistical analyses

Statistical analyses were performed via SPSS 26.0 (IBM Corporation) and SAS 9.4 (SAS Institute Inc.). Regarding the descriptive statistics, the continuous variables that were normally distributed are expressed as the means ± standard deviations; otherwise, the variables are represented by medians and quartiles, and the count data are expressed as rates. According to the specific data types and distribution characteristics, t-tests or nonparametric tests (Kruskal–Wallis H test) were used to analyze the discrepancies in the sociodemographic characteristics, medical conditions, and psychosocial variables between the SSD group and the non-SSD group. Univariate and multivariate linear regression analyses were used to explore the predictive role of SSD for QOL among breast cancer patients.

Mediation analyses were used in this study to examine the underlying relationship between SSD, psychological measures, and QOL. Two mediation models were used. Model 1 directly estimated the mediating effects of SSD between psychological measures and QOL without adjusting for any covariates. Model 2 estimated the mediating effects after adjusting for the following covariates: age, BMI, health insurance, residence, marital status, actual life situation, income, employment, activities in winter, activities in summer, smoking exposure, and alcohol exposure. Mediation analyses were conducted using the CAUSALMED procedure in SAS.

## 3. Results

### 3.1. Sociodemographic characteristics and medical conditions

**Figure 1:**
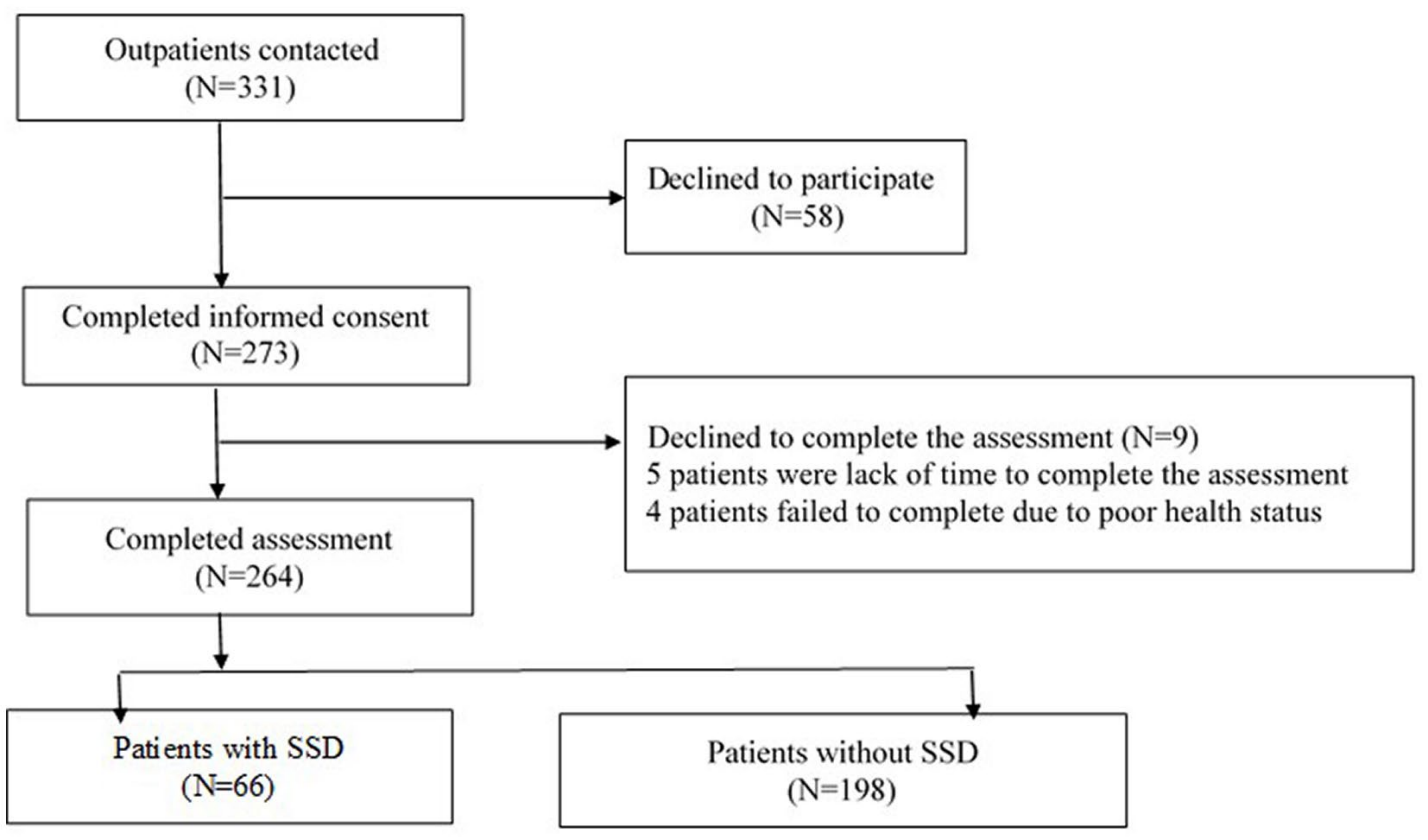
Study flowchart.

A total of 331 breast cancer patients were enrolled in this study, and 273 patients agreed to participate and signed informed consent forms. Nine patients withdrew due to a lack of time or poor health status. In total, 264 patients completed all the self-assessment questionnaires, with 66 patients (25.0%) being screened positive for SSD (see Figure 1 study flowchart). The sociodemographic characteristics and medical conditions are shown in Table 1. Furthermore, there were no significant differences in the sociodemographic characteristics between the patients with and without screened positive SSD. The patients with screened positive SSD had a lower performance status than those without SSD (t = 2.171, *p* = 0.031). In addition, more patients with screened positive SSD reported having received traditional Chinese medicine (TCM) treatments (*χ*^2^ = 5.046, *p* = 0.025) than patients without screened positive SSD.

**Table 1:** Sociodemographic characteristics and medical conditions.

Variables		SSD group (*n* = 66) M ± SD/*n* (%)	Non-SSD group (*n* = 198) M ± SD/*n* (%)	*t* ^a^*/χ*^2^	*P*
Age		50.94 ± 10.24	50.99 ± 10.20	0.035	0.972
BMI		23.14 ± 3.11	23.22 ± 3.24	0.157	0.876
KPS		90.15 ± 10.45	93.06 ± 9.05	2.171	0.031*
Residence	Urban	53 (80.3)	151 (76.3)	0.458	0.498
	Rural	13 (19.7)	47 (23.7)		
Health insurance	Yes	56 (84.8)	180 (90.9)	1.910	0.167
	No	10 (15.2)	18 (9.1)		
Marital status	Single/married but separated/divorced/widowed	9 (13.6)	20 (10.1)	0.630	0.427
	Married	57 (86.4)	178 (89.9)		
Living status	Living alone	4 (6.1)	13 (6.6)	0.021	0.885
	Living with others	62 (93.9)	185 (93.4)		
Education	Junior middle school and lower	15 (22.8)	42 (21.2)	0.424	0.515
	Senior middle school	12 (18.2)	53 (26.8)		
	University and above	39 (59.1)	103 (52.0)		
Family monthly income	8,000 RMB and lower	18 (27.3)	50 (25.2)	0.105	0.746
	Higher than 8,000 RMB	48 (72.7)	148 (74.8)		
Employment	Employed	23 (34.8)	85 (42.9)	1.332	0.248
	Unemployed/retired/housewife/others	43 (65.2)	113 (57.1)		
TNM stage	0	5 (7.6)	2 (1.0)	1.002	0.317
	1	14 (21.2)	20 (10.1)		
	2	11 (16.7)	40 (20.2)		
	3	35 (53.0)	42 (21.2)		
	4	65 (98.5)	89 (44.9)		
	Missing	1 (1.5)	5 (2.5)		
Surgery	Yes	51 (77.3)	163 (82.3)		
	No	15 (22.7)	35 (17.7)		
Chemotherapy	Yes	61 (92.4)	179 (90.4)	0.244	0.622
	No	5 (7.6)	19 (9.6)		
Radiation therapy	Yes	24 (36.4)	137 (69.2)	0.697	0.404
	No	42 (63.6)	61 (30.8)		
TCM therapy	Yes	20 (30.3)	34 (17.2)	5.046	0.025*
	No	46 (69.7)	162 (81.8)		
	Missing		2 (1.0)		

^a^Independent sample *t*-test; ^*^p < 0.05.

### 3.2. Comparison of psychosocial measures and QOL between patients with and without screened positive SSD

Compared to the non-SSD group, the screened positive SSD group had significantly higher levels of somatic symptoms, general anxiety, health anxiety, depression, fear of cancer recurrence, and negative illness perception, as well as lower QOL (*p* < 0.001; Table 2).

**Table 2:** Comparison of somatic symptoms and other psychological variables between patients with and without SSD.

Measures	M ± SD	SSD group (*n* = 66)	Non-SSD group (*n* = 198)	*t* ^a^	*p*
PHQ-15	5.81 ± 3.98	9.77 ± 3.68	4.49 ± 3.11	11.408	<0.001^***^
PHQ-9	5.93 ± 4.70	10.06 ± 4.17	4.56 ± 4.01	9.558	<0.001^***^
GAD-7	5.30 ± 4.34	9.32 ± 3.97	3.96 ± 3.56	10.277	<0.001^***^
WI-8	14.99 ± 6.07	21.95 ± 4.59	12.67 ± 4.54	14.355	<0.001^***^
FCR-4	6.62 ± 3.95	9.74 ± 4.29	5.58 ± 3.22	8.333	<0.001^***^
BIPQ-8	36.25 ± 12.17	44.12 ± 12.49	33.63 ± 10.89	6.527	<0.001^***^
FACT-B	101.17 ± 20.35	83.15 ± 13.37	107.17 ± 18.68	−9.651	<0.001^***^

^a^Independent sample *t*-test; ^***^p < 0.001.

### 3.3. The predictive role of screened positive SSD on quality of life of breast cancer patients

FACT-B scores (PWB, SWB, EWB, FWB, FACT-G-Total, FACT-B-TOI, and FACT-B-Total) were used as the dependent variables, and SSD-12 was entered as an independent variable in the linear regression analysis to explore the influence of SSD on QOL in breast cancer patients. The results are shown in Table 3. Screened positive SSD was a significant predictor of lower scores in all of the dimensions of the FACT-B, FACT-B-TOI, FACT-G-Total, and FACT-B-Total in breast cancer patients (*p* < 0.001). Additionally, a more severe presentation of screened positive SSD resulted in lower physical, social, emotional, and functional well-being, as well as more significant special concerns caused by breast cancer, thus resulting in lower QOL among breast cancer patients. After adjusting for the PHQ-15, PHQ-9, GAD-7, WI-8, FCR-4, and BIPQ-8 as covariates in the linear regression analysis, the SSD-12 still served as a strong predictor for lower physical (R^2^ = 0.0.833, *p* < 0.001), social/family (R^2^ = 0.555, *p* = 0.002), and functional well-being (R^2^ = 0.734, *p* = 0.002), as well as lower levels of BCS (R^2^ = 0.733, *p* = 0.003) and FACT-B-TOI (R^2^ = 0.876, *p* = 0.020).

**Table 3:** Predictive role of SSD (SSD-12) on QOL (dimensions of FACT-B) - results from univariate and multivariate linear regression analyses.

	R^2^	F	*p*	Unstandardized coefficients B	
				Constant	SSD-12
PWB univariate analysis	0.529	294.038	<0.001^***^	25.325	−0.476
Multivariate analysis	0.833	82.926	<0.001^***^	30.804	−0.201
SWB univariate analysis	0.062	17.435	<0.001^***^	22.993	−0.163
Multivariate analysis	0.555	16.294	0.002^**^	27.391	0.185
EWB univariate analysis	0.362	148.722	<0.001^***^	19.213	−0.304
Multivariate analysis	0.824	77.351	0.313	24.944	0.031
FWB univariate analysis	0.210	69.807	<0.001^***^	18.965	−0.283
Multivariate analysis	0.734	42.609	0.002^**^	24.784	0.140
BCS univariate analysis	0.414	185.051	<0.001^***^	29.049	−0.354
Multivariate analysis	0.733	42.362	0.003^**^	33.627	−0.120
FACT-B-TOI univariate analysis	0.525	289.156	<0.001^***^	73.338	−1.113
Multivariate analysis	0.876	133.983	0.020^*^	89.215	−0.181
FACT-G-total univariate analysis	0.396	171.639	<0.001^***^	86.496	−1.226
Multivariate analysis	0.870	114.207	0.137	107.923	0.155
FACT-B-total univariate analysis	0.466	228.230	<0.001^***^	115.544	−1.580
Multivariate analysis	00893	143.951	0.754	141.551	0.035

^*^p < 0.05; ^**^p < 0.01; ^***^p < 0.001. PWB, physical well-being; SWB, social/family well-being; EWB, emotional well-being; FWB, functional well-being; BCS, breast cancer subscale. FACT-B-TOI, FACT-B Trial Outcome Index (Score = PWB + FWB + BCS). FACT-G-Total, Functional Assessment of Cancer Treatment-General Scale Total (Score = PWB + SWB + EWB + FWB). FACT-B-Total, Functional Assessment of Cancer Treatment-Breast Cancer Total (Score = PWB + SWB + EWB + FWB + BCS). Each FACT subscale score was included into both univariate and multivariate linear regression analysis specifically. In univariate linear regression analysis, the SSD-12 was the independent variable, and all FACT subscales were dependent variables. All residuals were confirmed to be normally distributed. In multivariate linear regression analysis, SSD-12, PHQ-15, PHQ-9, GAD-7, FCR-4, WI-8, and BIPQ-8 scores were included as independent variables. The variance inflation factor (VIF) of the SSD-12 was 3.008.

### 3.4. Mediating effects of screened positive SSD between psychosocial measures and QOL

**Figure 2:**
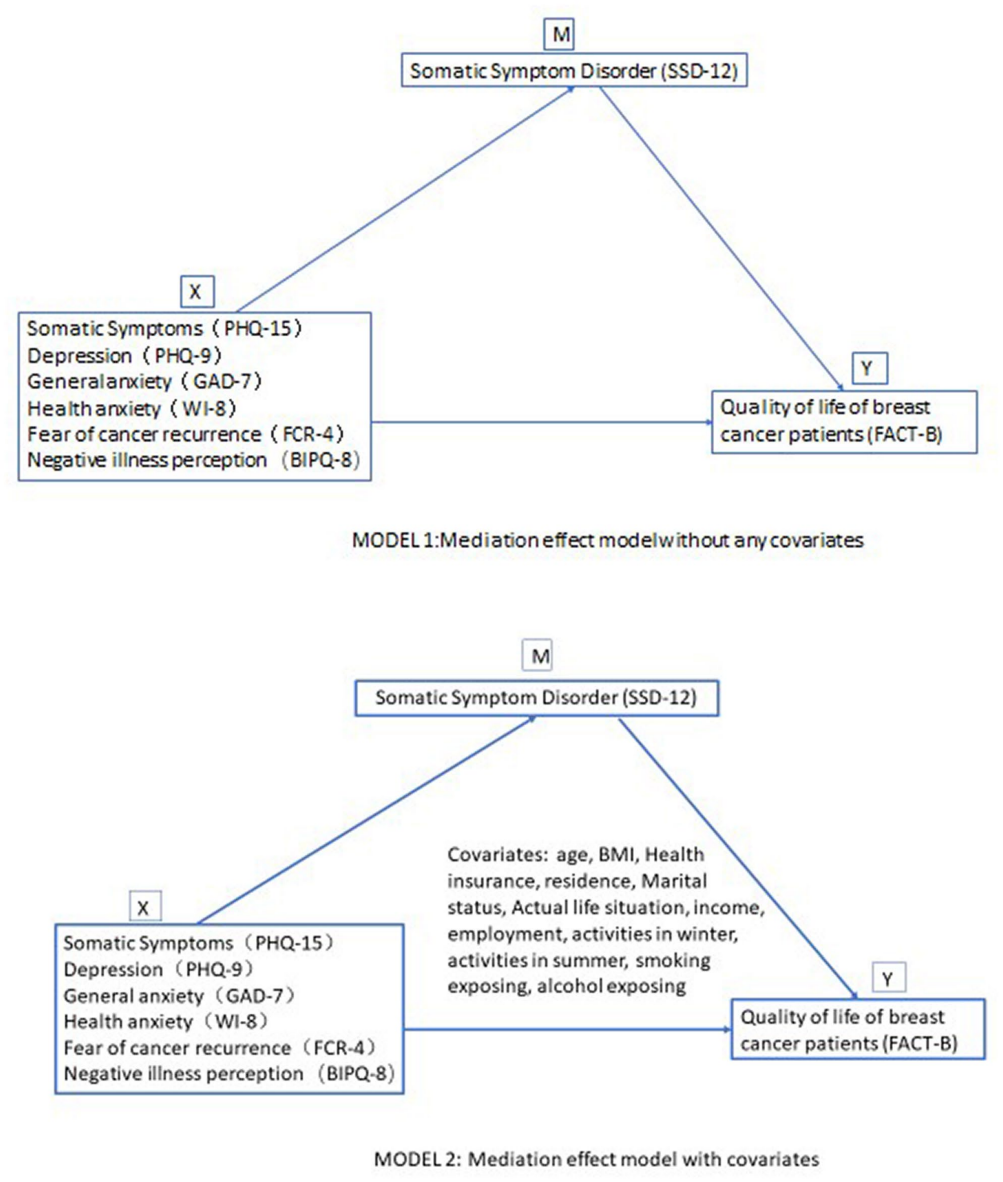
Models of the mediation effect of SSD.

Two mediation models were derived in the statistical analyses. In these models, somatic symptoms, general anxiety, depression, health anxiety, fear of cancer recurrence, and negative illness perception were independent variables; QOL was the dependent variable. Model 1 (without any covariates) showed that screened positive SSD had significant mediating effects between these psychological measures and QOL. The mediating effects ranged from the lowest of 25.668% (between depression and QOL) to the highest of 34.678% (between health anxiety and QOL). When incorporating covariates such as age, body mass index (BMI), health insurance, residence, marital status, living situation, income, employment, activities in summer or in winter, smoking exposure, and alcohol exposure in Model 2, significant mediating effects remained. The mediating effects ranged from the lowest of 26.836% (between depression and QOL) to the highest of 34.435% (between health anxiety and QOL). Detailed parameters are shown in Table 4; Figure 2.

**Table 4:** Two mediation effect models of SSD between psychosocial variables and quality of life in patients with breast cancer.

	Mediation effect (Model 1)					Adjusted mediation effect (Model 2)				
	Total effect (95% CI)	Natural direct effect (5% CI)	Natural indirect effect	Percentage mediated	Pr > |Z|	Total effect (95% CI)	Natural direct effect	Natural indirect effect	Percentage mediated	Pr > |Z|
PHQ-15	−0.734 (−0.816, −0.652)	−0.502 (−0.606, −0.398)	−0.232 (−0.309, −0.155)	31.599 (20.982, 42.217)	<0.0001	−0.748 (−0.832, −0.664)	−0.513 (−0.617, −0.408)	−0.235(−0.312, −0.158)	31.444 (21.104,41.785)	<0.0001
GAD-7	−0.760 (−0.839, −0.682)	−0.549 (−0.645, −0.452)	−0.211 (−0.281, −0.141)	27.809 (18.600, 37.019)	<0.0001	−0.754 (−0.833, −0.675)	−0.543 (−0.641, −0.444)	−0.211 (−0.283, −0.140)	28.019 (18.499,37.540)	<0.0001
PHQ-9	−0.778 (−0.854, −0.703)	−0.579 (−0.670, −0.488)	−0.200 (−0.265, −0.135)	25.668 (17.366, 33.970)	<0.0001	−0.759 (−0.836, −0.682)	−0.555 (−0.647, −0.463)	−0.204 (−0.270, −0.138)	26.836 (18.189,35.483)	<0.0001
WI-8	−0.716 (−0.800, −0.632)	−0.468 (−0.593, −0.343)	−0.248 (−0.347, −0.149)	34.678 (20.550, 48.807)	<0.0001	−0.722 (−0.807, −0.637)	−0.473 (−0.598, −0.349)	−0.249 (−0.347, −0.151)	34.435 (20.595,48.27)	<0.0001
BIPQ	−0.744 (−0.825, −0.664)	−0.526 (−0.613, −0.439)	−0.218 (−0.281, −0.156)	29.314 (21.242, 37.386)	<0.0001	−0.728 (−0.809, −0.646)	−0.510 (−0.597, −0.424)	−0.217 (−0.279, −0.155)	29.855 (21.684,38.025)	<0.0001
FCR-4	−0.729 (−0.812, −0.647)	−0.495 (−0.593, −0.398)	−0.234 (−0.305, −0.163)	32.051 (22.342, 41.761)	<0.0001	−0.735 (−0.818, −0.652)	−0.503 (−0.601, −0.405)	−0.232 (−0.303, −0.161)	31.523 (21.901, 41.144)	<0.0001

^***^P < 0.001. Model 1: Mediation effect model without any covariates. Model 2: Mediation effect model with covariates (age, BMI, health insurance, residence, marital status, living situation, income, employment, activities in summer or in winter, smoking exposure, and alcohol exposure).

## 4. Discussion

SSD in cancer has been recognized by psycho-oncologists. Grassi et al. suggested that special diagnostic criteria for SSD in cancer should integrate conventional psychiatric and psychosomatic criteria and that specific psychosocial interventions should be validated in cancer patients (26). Our results showed that the rate of screened positive SSD among breast cancer patients was 25.0%, which was lower than that in outpatients of general hospitals in China (33.8%) (27). This may indicate that the prevalence of SSD is divergent based on different populations; additionally, breast cancer patients may have more physical symptoms due to the disease itself and treatments, which may result in cancer patients and family members preferring to consider any discomfort as a physical symptom rather than a psychological issue. Moreover, patients can rebalance their health in a manner in which their health anxiety may be lower than that observed among patients in general hospitals (28). It is necessary to mention that this prevalence result came from the brief screening tool of the SSD-12. Although the SSD-12 has good reliability and validity, it cannot meet the diagnostic accuracy of psychiatric interviews. The SSD-12 results have clinical reference value, but they cannot be considered an SSD diagnosis. The descriptive results obtained from this study for SSD need to be interpreted appropriately. We found that a higher percentage of TCM exposure was reported in patients with SSD than in patients without SSD. In Chinese culture, an important role of TCM is “improving immunity.” Some cancer patients may choose TCM to improve their immunity and relieve symptoms, including somatic symptoms, while receiving anticancer treatments (29). As it is thought that cancer patients inevitably suffer from more severe symptoms, SSD is often ignored by oncologists and family caregivers. Therefore, we hope to present the phenomenon of SSD prevalence in breast cancer patients to highlight the need to pay more attention to this issue. The identification of SSD in breast cancer patients has both benefits and potential risks. The benefits are that more attention can be given to the psychological care of breast cancer patients with physical symptoms to help patients alleviate distress and improve their physical symptoms and quality of life. However, there are also some potential risks, such as that these physical symptoms may be caused by disease progression or anticancer therapies that might be ignored under the consideration of SSD. Therefore, we must be more careful when considering the diagnosis of SSD in cancer patients. Toussaint et al. suggested using combined scores of ≥23 in the SSD-12 and ≥ 9 in the PHQ-15 for psychiatric outpatients (30). However, Cao et al. suggested using DSM-V B criteria (SSD-12 or WI-8 alone) in Chinese general hospital settings (31). Cao et al. also suggested the use of a score ≥ 13 in the SSD-12 alone. Considering that the increasing sensitivity of a screening tool would both increase the potential negative risk of ignoring the symptoms caused by cancer and anticancer treatments and increase the screening burden in the oncology department, we finally used a score ≥ 16 in the SSD-12 suggested by Li^15^ in this paper.

The psychosocial measures for depression, anxiety, health anxiety, fear of cancer recurrence, and negative cognitive and emotional representations of disease in the patients with SSD were also significantly higher than those in the patients without SSD, thus indicating that symptom-related emotional distress is more prominent in the patients with SSD, which is similar to the results by other researchers (32, 33). Therefore, it is necessary to conduct a comprehensive assessment of psychological dimensions such as anxiety and depression, in addition to evaluating somatic symptoms. Furthermore, the QOL of the patients with SSD was significantly lower than that of the patients without SSD, which indicates that QOL in the patients with SSD was generally affected. Similar conclusions were obtained in a study on QOL in outpatients with SSD in general hospitals in China (34).

The current study found that screened positive SSD was a predictor of lower QOL in breast cancer patients; specifically, screened positive SSD interfered with all dimensions of QOL, as it caused lower physical, social, functional, and emotional well-being, as well as increasing specific concerns related to breast cancer. Additionally, we performed a multivariate linear regression analysis, which showed that even though confounding factors existed, SSD could also predict lower physical, social/family, and functional dimensions of QOL, as well as lower BCS and FACT-B-TOI scores. Other studies have also demonstrated a predictive role of somatic symptoms for health outcomes (11, 35). Therefore, specific care for SSD and the distress caused by SSD should be considered in high-quality cancer care to improve the QOL of cancer patients. The former study also indicated that psychological factors were associated with the QOL of breast cancer patients and suggested the incorporation of these factors in cancer care (36). Moreover, multidisciplinary interventions are recommended to improve QOL by reducing the distress caused by SSD and emotional problems (37).

Significant mediating effects of SSD were verified in this research, which suggested that SSD played an important role in the process by which psychosocial measures influence QOL for patients with breast cancer. Research on cancer patients with SSD is not sufficient. Most of the studies remain in the stage of presenting descriptive outcomes. More research on the mechanism of SSD in cancer, whether it has a profound impact on quality of life or survival, how to identify it properly and in a timely manner, and which kind of psychosocial interventions would be more effective are necessary in the future. This is the first study to explore how SSD affects quality of life in Chinese breast cancer patients. Both direct and indirect effects were verified in our sample, which emphasized the importance of identifying and caring for SSD among breast cancer patients. Patient-reported outcome (PRO) management, including common symptom management, has been integrated into oncology clinical guidelines or as a requirement for medical assessment by authorized organizations in many countries, as it has certainly influenced both quality of life and survival in cancer patients (38–40). SSD is the overlapping dimension of physical and psychological symptoms in the monitoring process among cancer patients, which indicates that clinicians should understand some physical symptoms in cancer patients from a psychological perspective and provide rational psychosocial care for patients with SSD.

### 4.1. Study limitations

Several limitations should be acknowledged. This was a cross-sectional study, which was unable to determine the causal relationship between SSD and QOL. We selected two breast cancer clinics, including one psychiatric clinic and one psychological clinic, where the patients may have more emotional and mental problems than in other clinics. Studies incorporating larger and more diverse cancer samples, as well as longitudinal studies, are recommended to further understand the relationship between SSD and QOL. Many cancer patients have more than one concomitant disease, especially advanced cancer patients who have received or are undergoing conventional anticancer treatments. However, we collected information on all somatic symptoms but did not distinguish between symptoms derived from cancer and those derived from treatments and those that resulted from concomitant diseases. We would like to consider this issue in future studies on SSD. Strict diagnostic criteria for SSD are needed and are preferable if the study purpose is to verify a confirmed diagnosis of SSD. Patient-reported outcomes (PROs) have been highlighted in oncological clinical trials (41) and integrated clinical cancer care (39), and distress reported by patients’ self-reported physical and psychosocial distress screening has been included in many clinical practice guidelines (42, 43). Swift and timely recognition of cancer patients’ distress with brief PRO measurements has been recommended as the first step. We would like to use the SSD-12 as the main measurement in our data analysis to indirectly verify the practicability of the utility of the SSD-12 among breast cancer patients. Additionally, interventional studies on SSD in cancer patients would provide more benefits to high-quality cancer care.

### 4.2. Clinical implications

SSD can be easily mistaken as a physical symptom among cancer patients, which results in both overmedication and insufficient psychosocial care. In our study, we demonstrated a significantly higher level of SSD among breast cancer patients, and its direct and indirect effects on QOL warrant greater attention to this unique issue among cancer patients.

### 4.3. Conclusion

Breast cancer patients with SSD had higher levels of somatic symptoms, general anxiety, health anxiety, depression, negative illness perception, fear of cancer recurrence, and lower QOL. SSD had both a direct negative influence (significant independent predictor of low QOL) and an indirect negative influence (strong mediation effect) on QOL among breast cancer patients.

## Data availability statement

The datasets presented in this article are not readily available because it is not allowed by the Regulations of the People’s Republic of China on the Management of Human Genetic Resources. Requests to access the datasets should be directed to the corresponding author for further application to the authorized Department of Management of Human Genetic Resources in China.

## Ethics statement

This study was approved by the Ethics Committees of Peking University Cancer Hospital (2019YJZ06). The patients/participants provided their written informed consent to participate in this study.

## Author contributions

LT supervised this study and revised this manuscript. YZ and ZL conducted this study and wrote this manuscript. YP, YH, LS, YW, and SH helped in the study process like collecting data and coordinators training. All authors contributed to the article and approved the submitted version.

## Conflict of interest

The authors declare that the research was conducted in the absence of any commercial or financial relationships that could be construed as a potential conflict of interest.

## Publisher’s note

All claims expressed in this article are solely those of the authors and do not necessarily represent those of their affiliated organizations, or those of the publisher, the editors and the reviewers. Any product that may be evaluated in this article, or claim that may be made by its manufacturer, is not guaranteed or endorsed by the publisher.
